# COMPARED EFFICACY OF INTRA-ARTICULAR INJECTION OF METHYLPREDNISOLONE AND TRIAMCINOLONE

**DOI:** 10.1590/1413-785220172505172581

**Published:** 2017

**Authors:** ABDUL FETTAH BUYUK, ERAY KILINC, ISMET YALKIN CAMURCU, SAVAS CAMUR, HANIFI UCPUNAR, ADNAN KARA

**Affiliations:** 1. Baltalimani Bone Diseases Research and Training Hospital, Istanbul, Turkey.; 2. Golhisar State Hospital, Burdur, Turkey.; 3. Medical Faculty, Erzincan University, Erzincan, Turkey.; 4. Umraniye Training and Research Hospital, Istanbul, Turkey.; 5. Medical Faculty, Istanbul Medipol University, Istanbul, Turkey.

**Keywords:** Osteoarthritis, knee, Injections, intra-articular, Methylprednisolone/administration & dosage, Triamcinolone, acetonide/administration & dosage

## Abstract

**Objective:**

To compare the effect of two different corticosteroid types in bilateral and symmetrical knee osteoarthritis (OA).

**Methods:**

One hundred and twenty-six patients received injections of methylprednisolone acetate (MP) in one knee and triamcinolone hexacetonide (TH) in the contralateral knee. Patients were evaluated before injection and 2, 4, 8, 12, and 24 weeks after.

**Results:**

Mean patient age was 68.5±9 years. Mean BMI was 26.3±2.6 kg/m^2^. At first admission, mean VAS score was 7.7±1.3 for the right side and 7.5±1.5 for the left side, and mean WOMAC score was 67.6±14.4. After bilateral intra-articular injection, VAS scores for both knees and WOMAC scores decreased significantly when initial scores were compared with 2, 4, 8, 12, and 24 weeks after injection (p<0.05). A statistically significant change was seen over time when VAS and WOMAC scores for 2, 4, 8, 12, and 24 weeks post-injection were compared to each other (p<0.05). No significant difference was seen between knee sides (p>0.05).

**Conclusion:**

MP and TH have similar efficacy in relieving pain and improving function. The efficacy of intra-articular corticosteroid injection peaks 2 weeks after injection and the effect continues until the 24^th^ week. ***Level of Evidence II, Comparative Prospective Study.***

## RESUMO

**Objetivo:**

Comparar o efeito de dois tipos de corticosteroides em osteoartrite (OA) de joelho bilateral e simétrica.

**Métodos:**

Cento e vinte e seis pacientes receberam injeções de acetato de metilprednisolona (MP) em um joelho e de triancinolona hexacetonida (TH) no joelho contralateral. Os pacientes foram avaliados antes da injeção e 2, 4, 8, 12 e 24 semanas depois.

**Resultados:**

A média de idade dos pacientes foi 68,5 ± 9 anos. O IMC médio foi 26,3 ± 2,6 kg/m^2^. Na primeira internação, o escore médio da EVA foi 7,7 ± 1,3 para o lado direito e 7,5 ± 1,5 para o esquerdo e a média do escore WOMAC foi 67,6 ± 14,4. Depois da aplicação bilateral das injeções intra-articular, os escores da EVA e do WOMAC para ambos os joelhos diminuíram significantemente ao comparar os escores iniciais com os de 2, 4, 8, 12 e 24 semanas depois da injeção (p < 0,05). Constatou-se diferença estatisticamente significante no decorrer do tempo, quando os escores EVA e WOMAC às 2, 4, 8, 12 e 24 semanas depois da injeção foram comparados entre si (p < 0,05). Não houve diferença significante entre os lados direito e esquerdo (p > 0,05).

**Conclusão:**

MP e TH têm eficácia similar quanto ao alívio da dor e à melhora da função. A eficácia da injeção intra-articular de corticosteroides atinge o máximo duas semanas depois da aplicação e o efeito continua até a 24^a^ semana. **Nível de Evidência II, Estudo Prospectivo Comparativo.**

## INTRODUCTION

Osteoarthritis of the knee is a major cause of pain and disability in older adults.^1^ Pain control is one of the main goals in treating knee OA.^2^ Management of this disease begins with conservative treatment such as physical therapy, exercise, weight loss, and medications; surgical intervention can be indicated for patients with advanced OA.^3^ Intra-articular corticosteroid injections (IACI) are frequently used and recommended by the American College of Rheumatology as part of conservative therapy for knee OA.^4^ The clinical benefits of IACI have been evaluated in several studies.^5^ ^-^ ^7^ Some studies have raised concerns about progression of cartilage destruction, but others have shown that corticosteroid injections can reduce this progression.^8^ ^,^ ^9^

The literature describes various inconsistent results from IACI; although some studies suggest short-term benefits (usually for one to four weeks), others suggest benefits may last up to 24 weeks.^10^ ^,^ ^11^ Some studies also have compared different types of corticosteroids for intra-articular injection. The perceived efficacy and rare adverse effects have made IACI a mainstay of knee OA management.^12^ ^,^ ^13^ Methylprednisolone acetate and triamcinolone hexacetonide are the most commonly used intra-articular corticosteroids.^14^

This present study consists of a randomized, prospective, multicenter investigation to determine the effect of two different types of corticosteroids on OA; this comparison was made by injecting bilateral and symmetrical knee joints involved with the two most commonly used compounds.

## MATERIAL AND METHODS

After written consent was obtained from all patients and approval by the institutional review board (process number 10840098-604.01,01-E.3351, 1/3/2016), 126 patients (101 female, 25 male) were included in the study. 

All patients presented to the outpatient orthopedic clinic with a bilateral knee pain score of ≥4 points on a 0-10 Visual Analog Scale (VAS) on the day of the examination. Patients were also required to have radiologically verified bilateral grade 3 OA of the knee according to the Kellgren-Lawrence classification.^15^ All patients in this study had dissatisfaction with previous attempts at conservative treatment including non-steroidal anti-inflammatory drugs. 

Exclusion criteria were: secondary arthritis, joint instability, IACI within the previous 6 months, history of diabetes mellitus, recent history of trauma to the knee, BMI >30, or presence of cancer or malignant disorders. Patients were also excluded if they had contraindications to injection, such as infection, anticoagulation therapy, allergy or hypersensitivity to any of the study medications. Patients who used systemic corticosteroids were also excluded, as were patients with a difference of >2 points between their knees on the VAS.

In this study we did not use a control group. Instead, we compared the medications by injecting methylprednisolone acetate (MP) in one knee and triamcinolone hexacetonide (TH) in the contralateral knee of the same patient. A randomization procedure was followed to assign each compound to the right or the left knee. 

Patients were placed in a sitting position with knee flexion of 90 degrees, and a lateral approach to the knee was used. The skin of the injection site was cleaned with povidone-iodine solution. No anesthetic was administered before injection. Either 1 mL of 40 mg methylprednisolone acetate (Depo-Medrol, Pfizer) mixed with 3 mL 1% lidocaine or 2 mL of 40 mg triamcinolone hexacetonide (Artropan, Kocak Farma, 20 mg/mL) mixed with 3mL 1% lidocaine was injected under sterile conditions using a 22G needle. Needles were changed between drawing up the steroid and injection.

Four orthopedic surgeons in three centers applied all of the injections. Additional injections were not permitted during the study period. 

A fifth surgeon who was not aware of the study design performed the clinical evaluation. Patients were evaluated before the injection and in control visits 2, 4, 8, 12, and 24 weeks after the injections. Pain severity was evaluated at each visit according to the VAS for each knee, and function was assessed using the WOMAC scale.^16^ Possible complications and side effects were also evaluated at each visit.

The statistical analysis was performed using SPSS 22.0 software (IBM Corp., Chicago, IL, USA). The Kolmogorov-Smirnov test was used to assess normal distribution of the variables. The non-parametric Wilcoxon test was used to compare VAS and WOMAC scores at first admission and at 2, 4, 8, 12, and 24 weeks after injection. The non-parametric Mann-Whitney U test was used to compare VAS scores for the right and left sides at 2, 4, 8, 12, and 24 weeks after injection. Analysis of variance (ANOVA) was used to compare repeated measurements of VAS and WOMAC scores at 2, 4, 8, 12, and 24 weeks after injection. 

## RESULTS

**Figure 1:**
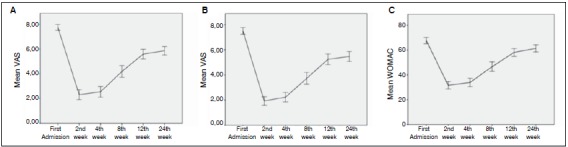
A: Linear graph showing mean VAS scores after injection with methylprednisolone acetate. B: Linear graph of mean VAS scores after injection with triamcinolone hexacetonide. C: Linear graph of mean WOMAC scores for both knees.

The mean age of the 126 patients was 68.5 ± 9 years (range: 57-83). Mean patient BMI was 26.3 ± 2.6 kg/m^2^ (range: 21-30). At first admission; mean VAS score was 7.7 ± 1.3 for right knees and 7.5 ± 1.5 for left knees, and mean WOMAC score was 67.6 ± 14.4. After bilateral intra-articular injection, there was a statistically significant decrease in the initial VAS scores for both knees and WOMAC score in comparison with these measurements taken 2, 4, 8, 12, and 24 weeks after injection (p<0.05). (Table 1) We also found a statistically significant change over time when VAS and WOMAC scores from 2, 4, 8, 12, and 24 weeks after injection were compared to each other (p<0.05), indicating that the pain relieving effect of both agents decreases over time. (Figure 1) No statistically significant difference was seen in VAS scores taken at first admission and 2, 4, 8, 12, and 24 weeks after injection when the right side (injected with methylprednisolone acetate) was compared with the left side (injected with triamcinolone hexacetonide). (p>0.05) (Table 1) 

**Table 1:** Mean VAS scores of right and left knee and mean WOMAC scores of the patients.

First admission*			2nd week*			4th week*		
VAS R	VAS L	WOMAC	VAS R	VAS L	WOMAC	VAS R	VAS L	WOMAC
7.7 ± 1.3	7.5 ± 1.5	67.6 ± 14.4	2.3 ± 2.2	1.9 ± 1.8	31.6 ± 17.3	2.5 ± 2.4	2.2 ± 2.1	33.9 ± 19.1
8th Week*			12th Week*			24th Week*		
VAS R	VAS L	WOMAC	VAS R	VAS L	WOMAC	VAS R	VAS L	WOMAC
4.1 ± 2.7	3.7 ± 2.6	46.6 ± 21.8	5.5 ± 2.2	5.2 ± 2.4	58.1 ± 18	5.8 ± 1.9	5.4 ± 2.2	61.3 ± 16.4

* ± standart deviation.

## DISCUSSION

Knee OA is a common degenerative joint disease and affects more than one-third of people over age 65.^17^ The most common presenting symptom of OA is pain. Two meta-analyses concluded that IACI is clinically and statistically effective in reducing pain.^10^ ^,^ ^18^ The exact mechanism by which intra-articular corticosteroid injection works is not yet clear, but it is thought that corticosteroids inhibit leukocyte secretion from the synovial cells and decrease synthesis of interleukins and prostaglandins.^17^ Synovial membranes in OA have been shown to be the source of proinflammatory cytokines that may be responsible for the clinical symptoms and progression of OA via cartilage destruction.^19^ A randomized, double-blind placebo controlled study by Raynauld et al.^20^ showed that long-term repetitive administration of IACI is effective for symptom relief and has no destructive effect on the anatomical structures of the knee.

Our study demonstrated that both intra-articular triamcinolone and methylprednisolone are effective at reducing pain and improving function in patients with knee OA, and their efficacy may last up to 24 weeks. In this study we observed that for patients who benefited from intra-articular injection, both steroid types had similar effects and duration of efficacy. 

There are studies comparing corticosteroid types in the literature. Pyne et al.^21^ reported that triamcinolone was statistically more efficient in pain relief 3 weeks after injection than methylprednisolone. In another study, however, Yavuz et al.^22^ stated that methylprednisolone was statistically more effective in relieving pain than other agents including triamcinolone until 6 weeks after injection. In our own study, no difference was observed between the two types of corticosteroids in terms of pain relief.

Although it is administered locally, a significant portion of the active corticosteroid compound may be absorbed from the joint into the circulation and result in systemic effects. Most of studies in the literature evaluated the hypothalamic-pituitary-adrenal axis. Serum cortisol levels decrease within hours of injection and usually return to recovery level in 1 to 4 weeks, but this may take longer depending on the type and dose of IACI.^23^ The most common dose preference for the knee joint varies from 20 to 80 mg methylprednisolone or 20 to 40 mg triamcinolone.^10^ ^,^ ^24^ We used 40 mg triamcinolone and 40 mg methylprednisolone. The most severe complications of IACI are septic arthritis and steroid-induced arthropathy,^25^ but the complications are rare.^26^ In our study, 19 of 126 patients had mild pain at the injection site which subsided in a day; no patients had any significant adverse effects. 

This study was limited by the fact that we investigated only two types of corticosteroids. We investigated the most commonly used agents; other types could yield different results. Another limitation is the use of the VAS, an objective test for evaluating outcomes.

## CONCLUSION

In conclusion, bilateral IACI using either methylprednisolone or triamcinolone is safe and effective at reducing pain in patients with bilateral knee OA. Both intra-articular triamcinolone and methylprednisolone have similar efficacy in relieving pain and improving function. The efficacy of IACI is highest 2 weeks after injection and the effect continues to 24 weeks after injection. 
